# Diabetic peripheral neuropathy class prediction by multicategory support vector machine model: a cross-sectional study

**DOI:** 10.4178/epih.e2016011

**Published:** 2016-03-24

**Authors:** Maryam Kazemi, Abbas Moghimbeigi, Javad Kiani, Hossein Mahjub, Javad Faradmal

**Affiliations:** 1 Department of Biostatistics, School of Public Health, Hamadan University of Medical Sciences, Hamadan, Iran; 2 Modeling of Noncommunicable Diseases Research Center, Hamadan University of Medical Sciences, Hamadan, Iran; 3 Department of Endocrinology, College of Medical Sciences, Hamedan University of Medical Sciences, Hamedan, Iran; 4 Department of Internal Medicine, College of Medical Sciences, Hamedan University of Medical Sciences, Hamedan, Iran; 5 Research Center for Health Sciences and Department of Biostatistics, School of Public Health, Hamadan University of Medical Sciences, Hamadan, Iran

**Keywords:** Support vector machine, Diabetic neuropathy, Classification, Logistic models

## Abstract

**OBJECTIVES:**

Diabetes is increasing in worldwide prevalence, toward epidemic levels. Diabetic neuropathy, one of the most common complications of diabetes mellitus, is a serious condition that can lead to amputation. This study used a multicategory support vector machine (MSVM) to predict diabetic peripheral neuropathy severity classified into four categories using patients’ demographic characteristics and clinical features.

**METHODS:**

In this study, the data were collected at the Diabetes Center of Hamadan in Iran. Patients were enrolled by the convenience sampling method. Six hundred patients were recruited. After obtaining informed consent, a questionnaire collecting general information and a neuropathy disability score (NDS) questionnaire were administered. The NDS was used to classify the severity of the disease. We used MSVM with both one-against-all and one-against-one methods and three kernel functions, radial basis function (RBF), linear, and polynomial, to predict the class of disease with an unbalanced dataset. The synthetic minority class oversampling technique algorithm was used to improve model performance. To compare the performance of the models, the mean of accuracy was used.

**RESULTS:**

For predicting diabetic neuropathy, a classifier built from a balanced dataset and the RBF kernel function with a one-against-one strategy predicted the class to which a patient belonged with about 76% accuracy.

**CONCLUSIONS:**

The results of this study indicate that, in terms of overall classification accuracy, the MSVM model based on a balanced dataset can be useful for predicting the severity of diabetic neuropathy, and it should be further investigated for the prediction of other diseases.

## INTRODUCTION

Diabetes is growing at epidemic levels worldwide. In 2013, 385 million people worldwide had diabetes, and by 2035, the number is expected to increase to 539 million [1]. In Iran, 8.4 percent of the total population over 20 years of age had diabetes in 2013, and it is estimated that the prevalence will reach 12.3% in 2035 [2].

Patients with diabetes may experience a wide range of neurological disorders that can involve different types of sensory and motor nerves. Diabetic peripheral neuropathy (DPN) is one neuro muscular disorder that can occur in patients with diabetes [3]. This usually occurs within 10 years of the onset of the disease in 40% to 50% of patients. People with type 1 diabetes may not experience neuropathy for up to five years, but in patients with type 2 diabetes, complications may exist from the onset of the disease [4]. Diabetic foot ulcers are largely dependent on the sensory impairment caused by diabetic neuropathy, which is a serious complication that can lead to amputation. Other problems in diabetic neuropathy and neuropathic pain are caused by dysfunction of the sympathetic nervous system and can cause many problems for the patient and the therapist [5]. Early neuropathy detection and prediction is of great importance in the prevention of complications such as pain, loss of sensation, foot ulcers, gangrene, and amputations.

In previous studies, the global prevalence of painful DPN has been reported to be from 23% to 54% of cases of diabetes [6]. Estimates of the prevalence of diabetic neuropathy vary greatly due to differences in the types of patients, neurological complications of aging, and diagnostic criteria. To our knowledge, no studies have examined the prevalence of DPN in the different geographical areas of Iran. The prevalence of diabetic neuropathy has been reported to vary from 16% to 87%, and the overall prevalence of DPN was estimated at 53% of the population with diabetes [7].

Recently, various machine learning methods have been applied to disease classification. One study used a K-nearest neighbors (KNN) algorithm, random forest model, and a support vector machine (SVM) with a linear kernel function and radial basis function (RBF) to classify the different levels of airway obstruction in patients with chronic pulmonary diseases in a binary fashion [8]. A binary SVM classifier was used for the identification of schizophrenia spectrum disorders (SSD) in the early stages and the assessment of the predictive value of early diagnosis of different types of data in the emergence of first episode psychosis (FEP) [9]. Another study has been conducted on the methods of diagnosis of lymphglands based on SVM with different kernel functions such as linear, quadratic, and Gaussian functions [10]. To predict the risk of complications with drug eluting stents (DES) in another study, an SVM model was used to classify patients under going DES placement in to a high and a low risk category, and to solve the problem of class imbalances, synthetic minority oversampling techniques were used to obtain a better performance with the unbalanced dataset [11]. In one study, mega-trend diffusion (MTD) has been used to increase minority class examples and balance in the dataset. At the prediction level, machine learning methods such as KNN and SVM were used to predict the presence of breast colon cancer patients in the dataset [12].

No study has yet used a multicategory support vector machine (MSVM) model to predict the severity of diabetic neuropathy with an unbalanced dataset. The aim of a this study is to predict DPN severity by using patients’ demographic characteristics and clinical features to classify patients into four categories: healthy, mild neuropathy, moderate neuropathy, and severe neuropathy.

Since identifying the onset of neuropathy is difficult and it manifests differently for each patient, and the diagnostic criteria for this disease remain unclear, classification and diagnosis of the patient into a particular disease severity level based on demographics and clinical characterization could prevent terrible complications, such as amputation, from neuropathy that would have otherwise been discovered at a more severe stage; thus machine learning as a tool for helping diagnose DPN and determining a prognosis could be very useful.

## MATERIALS AND METHODS

### Data source

In this study we used data collected at the Diabetes Center of Hamadan. In the province of Hamadan, this center is the only center for activities such as admitting patients with diabetes for treatment and providing preventive care for diabetes complications. In addition, this center carries out diabetes research. A convenience sampling method was used to enroll patients. Patients were matched with the American Diabetes Association criteria for diabetes type 1 “due to β-cell destruction, usually leading to absolute insulin deficiency” and diabetes type 2 “due to a progressive insulin secretory defect on the background of insulin resistance” [13]. Six hundred people were recruited, from April 18 to September 4, 2011. After obtaining informed consent, a questionnaire was administered containing general information, height, weight, smoking status, duration of the disease, medications, history of foot ulcer and laser photocoagulation. Then clinical characterizations were recorded. Patients were examined for DPN by an endocrinologist. The neuropathy symptoms score (NSS) and neuropathy disability score (NDS) were used as the criteria for diagnosing diabetic neuropathy. Then an NDS information quastionaire was administered. This information sheet contains parameters such as ankle reflexes and perceptions of needles, cold, and vibration. A score of zero to two is assigned to each parameter. In this study, the NDS score was used to classify severity of the disease. NDS≤2 was considered without neuropathy, 3≤NDS≤5 mild neuropathy, 6≤NDS≤8 moderate neuropathy, and NDS≥9 severe neuropathy [14].

### Analysis methods

The SVM was introduced for the first time in 1992 by Vapnik [15] as a new method for solving problems of classification and regression. A support vector classifier is an extension of a maximum margin classifier. Where the data cannot be separated and the classes overlap, we use a support vector classifier, which is also called a soft margin classifier. When observations are not linearly separable, we enlarge the input feature space to a higher dimensional feature space in which the data are separable.This method is called a SVM [16]. Suppose we have a dataset with n observations {x_i_,y_i_}, i=1,2,….*n*. When x_i_ ϵ*R^d^* and y_i_ ϵ{-1,1}, the objective function is min $\frac{1}{2}$ *w^t^w* where y_i_ (*w^t^*x_i_+b)≥1-ξ_i_ and ξ_i_ ≥0. When the data overlap, penalties are considered for data on the wrong side. The degree of violation of any piece of data is shown with ξ where ξ ≥0. Any data with ξ_i_ must pay a penalty. C is the penalty parameter that balances model complexity and misclassification.We look for the solution with ξ_i_ ≥0 as follows:

$$\min\frac{1}{2}w^{t}w\;+\;C\sum_{i}\xi_{i}$$

For the SVM for non-linear classification where linear separation is not possible, we take mapping points from the input space x to a higher dimension feature space using kernel functions and we create a separatorin a new space; the non-linear separator becomes:

$$y\;=\;sign(\sum_{i}\alpha_{i}y_{i}k(x_{i},\;x)+b)$$

Where k(.) is the kernel function for various types, such as kernel polynomial, RBF or Gaussian kernel, linear, or tangent [17].

SVM is generally used for two-class classification, but can also be used for multiple-class classification. Two simple methods for doing this are the strategies of “one-against-all” (OAA) and “one-against-one” (OAO). In fact, C classes convert a set of binary classification problem and generate several classifiers [16]. In In OAA, C binary SVMs are constructed to classify C classes that separate the desired class from the rest of the classes. For the final decision, a winner-take-all strategy is used [15,18]. In OAO, we have $\frac{C(C-1)}{2}$ binary classifiers. For the final decision, a maxwins voting strategy is used [18,19].

When classes are unbalanced, that is, the number of observations in each class differs dramatically, the SVM has a bias toward the majority classes and performs poorly for the classes with a small number of members. To overcome this problem, several preprocessing algorithms have been developed [20]. One of these algorithms is an oversampling approach and synthetic data generation method known as the synthetic minority class oversampling technique (SMOTE). There are different ways to resample the dataset, one of which is oversampling a minority class or undersampling a majority class. The SMOTE algorithm is a special case of oversampling to produce synthetic examples and can be used with a combination of the undersampling method [21, 22].

To avoid over-fitting, 10-fold cross-validation was used to compare different models on datasets. In each validation, nine sets of the original data were used to train the classifier and one set of data is classified. For SMOTE dataset, the data were balanced using SMOTE and were used as a training classifier. Randomly, 75% and 25% of the data were divided into two groups: training and testing, respectively. Machine learning algorithm performance is typically assessed using the accuracy of the prediction and precision of each class as:

$$\mathrm{Accuracy}\;=\frac{\mathrm{The}\;\mathrm{number}\;\mathrm{correctly}\;\mathrm{classified}}{\mathrm{Total}\;\mathrm{number}}\;{\mathrm{Precision}}_{i}\;=\frac{\mathrm{The}\;\mathrm{number}\;\mathrm{correctly}\;\mathrm{predicted}\;\mathrm{in}\;\mathrm{ith}\;\mathrm{class}}{\mathrm{Total}\;\mathrm{number}\;\mathrm{of}\;\mathrm{ith}\;\mathrm{class}}$$

In this study, we used the MSVM model with both OAA and OAO methods and three kernel functions—RBF, linear and polynomial—to predict the severity class of disease with the unbalanced dataset and used the SMOTE algorithm to improve the model performance. We used the mean accuracy to compare the performance of the models.

The SMOTE algorithm can be run with three parameters; one of the parameters is the number of nearest neighbors (k) that is used to generate the new examples of the minority class. The percent of oversampling (perc.over) is another parameter that determines how many extra cases of the minority class are generated. The number that drives the decision of how many extra cases from the majority classes are selected for each case generated from the minority class is the percent of undersampling (perc.under).

To select the best percentage of oversampling and undersampling to use in order to construct the optimal SMOTE dataset, the K-fold cross-validation was utilized. The minority class was oversampled at percentages from 100% to 1,000%, and the percentage of undersampling varied from 100% to 1,000%. While the precision of each class was satisfactory and our dataset was balanced, the optimal settings for SMOTE related to the percentage of oversampling and mean accuracy of the minor and major classes. In our study, the best parameters found were k= 5, perc.over=300, and perc.under=500.

We performed this study using R, an open-source statistical software language. Packages were used to regularize the kernel function and the penalty parameters (e1071), to use OAO-SVM and OAA-SVM (KlaR), to balance the dataset (DMwR) and for feature selection (Boruta).

## RESULTS

The patients’ age (mean±standard deviation) was 53.26± 14.46 years and mean disease duration was 9.26±7.44 years. In our study, there were 175 patients without neuropathy, 265 patients with mild neuropathy, 127 patients with moderate neuropathy, and 33 patients with severe neuropathy. Among the 20 features recorded for each patient, 13 features were finally selected using the Boruta algorithm, and all classifiers were built with these selected features. The output of the Boruta algorithm is a subset of the feature sets with the “important” label, with the measure of feature importance indicated by the Z-score. We chose the “important” feature sets and ordered the feature sets by the mean of importance. After fixing the tentative attributes, the selected features were age, type of diabetes, education level, BMI, history of blood pressure, systolic blood pressure, history of foot ulcer, medications, weight, history of laser photocoagulation, duration, average blood glucose, and height. These features were ordered by the mean of importance. The general characteristics of the study population are shown in Table 1.

The performance of multi-class classification for severity of diabetic neuropathy using MSVM with OAO and OAA strategies is shown in Table 2. To improve the performance of the model and balance the dataset we used the SMOTE algorithm. The classification was done using three kernel functions: RBF, linear, and 3-degree polynomial. The kappa coefficients of agreement for all classifiers have been determined in Table 2. The kappa agreement coefficients for classifiers that were built on the balanced dataset were between 0.6 and 0.8. This means that the agreement between observation and prediction was substantial with these classifiers [23].

Finally, the precision of the three most remarkable classifiers in each of the four diabetic neuropathy classes is shown in Table 3. The classifier ultimately chosen for predicting DPN classes is SMOTE-MSVM with the OAO strategy using the RBF kernel. The precision of prediction of each class is compared with multicategory logistic regression in Table 3.

## DISCUSSION

In this article, using an MSVM model with two strategies, OAO and OAA, we predicted neuropathy disease severity classes in patients with diabetes in Hamadan province, Iran in four categories: no neuropathy, mild neuropathy, moderate neuropathy, and severe neuropathy. The results showed that the classification in the case of the RBF kernel function and OAO strategy produced the best average accuracy.

The accuracy of classifiers built based on an unbalanced dataset is much less than the classifiers built on a balanced dataset. The SMOTE algorithm was used for oversampling of the severe neuropathy class and the undersampling method was used for the other classes which balanced the dataset among all the four categories. The results showed that the SMOTE approach can improve the accuracy of the classification of the minority class.

In the unbalanced dataset, the highest accuracy was achieved with the RBF kernel function, followed by the linear kernel function, and finally the polynomial kernel function with degree three. However, in the case of the balanced dataset, the polynomial kernel function worked a little better than the linear kernel function. The RBF kernel resulted in the lowest classification error, and the kappa coefficient confirmed this trend. The accuracy of classifiers when the OAO strategy was used was higher than with the OAA strategy for both datasets—unbalanced and balanced. The accuracy and kappa coefficients showed that MSVM can be useful for class prediction of the severity of diabetic neuropathy. The best classifier in our study was MSVM on a balanced dataset with the OAO method using the RBF kernel; the kappa coefficient of this classifier was found to be 0.68. On the balanced dataset, the kappa values were moderate but substantial, but in the imbalanced dataset, the values were only fair. These kappa values showed that the classifiers built on the balanced dataset were more valuable.

In most studies, the MSVM is used with microarray data and high-dimensional data. Although this model can be used for other data as well, with a balanced dataset, the result is more favorable. For predicting classes of diabetic neuropathy, we found that it was better to use a classifier built based on a balanced dataset and use the RBF kernel function and OAO strategy. This classifier correctly predicted the patient class in about 76% of cases. According to the results (Table 3), when the classes were imbalanced, logistic regression had good prediction, but only for the majority class, and MSVM in the moderate class worked slightly better than logistic regression, but in the minority class both of the classifiers performed poorly and actually could not predict the minority class correctly. According to the confusion matrices using classifiers built on the imbalanced dataset, class prediction precisions of the normal and mild diabetic neuropathy were better than for the moderate and severe classes, and distinguishing between the mild and moderate classes was difficult. Most moderate-risk patients were incorrectly classified in to the mild class.

In a study conducted on the same data, Kiani et al. [24] investigated the prevalence of diabetic neuropathy in Hamadan and detected the risk factors for diabetic neuropathy using multiple logistic regression. The significant variables from logistic regression and the features selected using the feature selection algorithm in our study are the same.

Duckstein et al. [25] conducted a study on discriminant analysis of the level of severity of diabetic neuropathy categorized by somatosensory-evoked potentials. Their study was carried out on 91 patients with diabetes mellitus. The target variable represented three groups: without neuropathy, mild neuropathy, and severe neuropathy. There were 26 explanatory variables; after discriminant analysis, 14 variables were identified to play a substantial role in identification of the classes and distinctions between them. However, in their study, a model to predict the occurrence and severity of neuropathy was not provided.

Duckstein et al. [25] classified diabetic neuropathy patients based on an invasive electrophysiological test, using fuzzy set structures. This test requires special equipment, knowledge, and professional training and cannot be widely used in the public health system. Although this test is very reliable, neuropathy is not otherwise detectable at an early stage of the disease.

In a study recently performed by Picon et al. [26] on 50 patients with diabetes, the severity of neuropathic disease was classified into three categories: mild, moderate, and severe neuropathic levels using a fuzzy model. This model was based on the experience and knowledge of experts in diabetic neuropathy and four input variables, including symptom assessment, sign examination, duration of disease, and HbAc1 were used. In our study, we used duration and A1c (average blood glucose), and other demographic and clinical characteristics to classify the severity of disease. NDS scores were used to determine the class of the target variable and NSS scores were not used for prediction. However, adding this variable to the model could increase the accuracy of the model [26].

Using this classifier alongside demographic and clinical characteristics of patients with diabetes, it was possible to predict the class of diabetic neuropathy a patient belonged to. The MSVM classifier performed better than a multicategory logistic regression model (accuracy=0.57) for predicting diabetic neuropathy, especially in the prediction of the normal and moderate classes. In an unbalanced dataset, the mean accuracy of the logistic and MSVM models were almost identical, but the logistic model better predicted the members of the class with larger sample sizes. After using SMOTE-MSVM with oversampling of the minority class to create synthetic data and using a balanced dataset, prediction was satisfactory in all classes. The final chosen classifier for predicting DPN classes was SMOTE-MSVM with the OAO strategy using the RBF kernel. Its precision in predicting each class compared with multicategory logistic regression is shown in Table 3. These results show that the prediction of minority classes is greatly improved by using a classifier built on a balanced dataset.

The current study had some limitations. It was possible that our patients were not representative of all patients with diabetes, since this study was performed in one center. Furthermore, the patients’ glycemic control was not clear either. Finally, we did not evaluate other causes of neuropathy.

For future studies with unbalanced datasets, we suggest that instead of using algorithms such as SMOTE which generate synthetic data and make changes in the original datasets, alternative methods such as the fuzzy SVM model be used.

The MSVM model is useful for identifying patients who could benefit from immediate intervention during the early stages of neuropathy to maintain the patient’s quality of life. Using this model for diabetic neuropathy severity prediction for patients with diabetes, treatment of patients prone to mild neuropathy should be different than the treatment of patients prone to severe neuropathy. For the patients in the severe stage, blood sugar, blood fat, and blood pressure should be controlled, and a greater focus on foot ulcers, kidneys and ophthalmic problems is needed as well. This model can be implemented in any public health system and may be an important tool to prevent the complications of disease progression.

## Figures and Tables

**Table 1.** Baseline characteristics of the study population by severity of DPN

Feature	DPN			
	No	Mild	Moderate	Severe
Cases of DPN	175 (29.2)	265 (44.2)	127 (21.2)	33 (5.5)
Age (yr)	43.98±15.20	54.7±12.02	61.91±11.20	62.67±10.27
A1c	8.45±1.00	8.82±1.20	9.07±6.26	8.53±0.80
Creatine	0.98±0.23	1.01±0.39	1.06±0.35	1.09±0.30
Cholesterol (mg/dL)	182.2±34.22	189.49±37.78	180.44±31.77	185.10±29.81
HDL	43.94±9.25	42.49±8.49	44.17±32.79	42.51±7.24
LDL	105.6±25.75	111.66±29.61	105.47±25.43	108.08±23.29
Triglycerides (mg/dL)	163.20±89.39	179.70±112.79	173.56±75.54	172.99±80.51
Duration (yr)	6.78±5.93	9.12±7.21	11.78±8.46	13.66±7.20
BMI (kg/m^2^)	26.25±4.10	29.32±12.47	28.45±4.66	26.29±4.00
Weight (kg)	66.99±11.90	71.93±11.98	73.10±12.89	68.53±10.74
Height (cm)	159.74±9.28	158.34±10.53	160.35±8.76	161.66±10.01
Systolic blood pressure (mm Hg)	117.43±18.06	123.91±19.09	129.57±20.13	133.33±22.45
Diastolic blood pressure (mm Hg)	66.77±11.58	70.15±10.67	71.85±13.04	73.94±12.73
Education level				
Illiterate	33 (18.9)	80 (30.2)	59 (46.5)	17 (51.1)
Elementary	24 (13.7)	81 (30.6)	21 (16.5)	4 (12.1)
<High school	88 (50.3)	83 (31.3)	41 (32.3)	10 (30.3)
≥High school	30 (17.1)	21 (8.0)	6 (4.8)	2 (6.0)
History of hypertension				
No	140 (80.0)	160 (60.4)	50 (39.4)	10 (30.3)
Yes	35 (20.0)	105 (39.6)	77 (60.6)	23 (69.7)
Smoking				
No	29 (87.9)	245 (92.5)	117 (92.1)	29 (87.9)
Yes	4 (12.1)	20 (7.5)	10 (7.9)	4 (12.1)
Medications				
Oral agent^1^ (baseline=1)	97 (55.4)	149 (56.2)	59 (46.5)	12 (36.4)
Insulin	62 (35.4)	74 (27.9)	43 (33.9)	12 (36.4)
Oral agent + insulin	16 (9.1)	43 (15.8)	25 (19.7)	9 (27.3)
History of foot ulcer				
No	168 (96.0)	238 (89.8)	101 (79.5)	20 (60.6)
Yes	7 (4.0)	27 (10.2)	26 (20.5)	13 (39.4)
History of laser photocoagulation (%)				
No	170 (97.1)	238 (89.8)	99 (78.0)	18 (54.5)
Yes	5 (2.9)	27 (10.2)	28 (22.0)	15 (45.5)
History of foot ulcer				
No	168 (96.0)	238 (89.8)	101 (79.5)	20 (60.6)
Yes	7 (4.0)	27 (10.2)	26 (20.5)	13 (39.4)
Type of diabetes				
Type 1	49 (28.0)	25 (9.4)	4 (3.1)	1 (3.0)
Type 2	126 (72.0)	240 (90.6)	123 (96.9)	32 (97.0)

Values are presented as mean±standard deviation or number (%). DPN, diabetic peripheral neuropathy; A1c, glycated hemoglobin; HDL, high-density lipoprotein; LDL, low-density lipoprotein; BMI, body-mass index.

1 Oral agents: metformin, glibenclamide, gliclazide, pioglitazone.

**Table 2.** The mean accuracy of MSVM with OAO and OAA strategies

Method	Kernel function	Accuracy of SVM unbalanced dataset	Accuracy of SMOTE-SVM	Kappa coefficient
OAA	RBF	0.51	0.67	0.52
	Polynomial	0.46	0.65	0.49
	Linear	0.47	0.64	0.47
OAO	RBF	0.53	0.76	0.68
	Polynomial	0.46	0.73	0.60
	Linear	0.54	0.72	0.59

MSVM, multicategory support vector machine; OAO, one-against-one; OAA, one-against-all; SVM, support vector machine; SMOTE, synthetic minority class oversampling technique; RBF, radial basis function.

**Table 3.** The precision of each diabetic neuropathy classification with three classifiers

Classifiers	DPN			
	No	Mild	Moderate	Severe
SMOTE-OAO-MSVM	0.73	0.71	0.85	0.80
OAO-MSVM^1^	0.69	0.64	0.46	0.10
Multicategory logistics	0.55	0.73	0.39	0.10

DPN, diabetic peripheral neuropathy; SMOTE, synthetic minority class oversampling technique; OAO, one-against-one; MSVM, multicategory support vector machine.

1 Radial basis function kernel using MSVM models.
